# Insights from a community-based palliative care course: a qualitative study

**DOI:** 10.1186/s12904-021-00769-4

**Published:** 2021-07-13

**Authors:** Tania Pastrana, Johannes Wüller, Simone Weyers, Eduardo Bruera

**Affiliations:** 1 Department of Palliative Medicine, Medical Faculty, RWTH Aachen University, Pauwelsstr. 30, 52074 Aachen, Germany; 2 Home Care Städteregion Aachen Non-profit Limited Liability Company, Aachen, Germany; 3 Institute of Medical Sociology, Centre for Health and Society (CHS), Düsseldorf University Hospital, Düsseldorf, Germany; 4 Department of Palliative, Rehabilitation and Integrative Medicine, The University of Texas MD Anderson Cancer Center, Houston, TX USA

**Keywords:** Community-based learning, Palliative care, Education, Undergraduate, Qualitative study, Focus group

## Abstract

**Background:**

The vast majority of medical students have no exposure to clinical palliative care encounters, especially in the community. Medical schools should respond to current challenges and needs of health systems by guaranteeing students adequate training that addresses palliative care needs of populations in different settings. The main purpose of this qualitative study was to capture the experiences of a select group of medical students’ following a community-based PC course.

**Methods:**

We carried out a qualitative study using two focus groups to capture the experience of medical students in a course that combined classroom teaching with community-based learning for undergraduate medical students in Germany. Discussions were transcribed and analyzed thematically.

**Results:**

Fifteen female students in their 2nd to 5th year participated in the focus groups, which provided didactic teaching and experiential learning. Four areas were particularly relevant: (1) authenticity, (2) demystification of the concepts of palliative care through personal contact with patients, (3) translation of theoretical knowledge into practice, and (4) observation of a role model interacting with seriously ill patients and engaging in difficult conversations.

**Conclusion:**

Students whose encounters with patients and their families went beyond a review of their medical records had a better grasp of the holistic nature of PC than those who did not. Bringing students directly from the hospital to patients in their homes reinforced the benefits of an integrated healthcare system.

## Background

More people will live longer with more chronic illnesses in coming decades. Their preferred place of care and death is their home [1, 2]. This preference challenges both healthcare systems and informal caregivers, especially when it comes to delivery of palliative care (PC) services. Medical and allied health schools must respond to this growing need [3]. Although PC is gradually being integrated into medical school and allied health curricula, it requires exposing clinical trainees to the reality of chronic illness, which means teaching students patient care in the community [4, 5]. This exposure has been found to improve newly qualified doctors’ competence and confidence in delivering care to this patient group [6].

Community-Based Learning (CBL) based on experience, supported by guidance, contextualization, knowledge, and analysis is sustainable learning. Immersion in practice during the learning process is the most effective method of incorporating attitudes and skills that didactic methods alone cannot convey [7]. PC training in patients’ homes offers students the opportunity to learn in patients’ home settings, providing a window to their personal lives and families, as well as the impact of culture and environment on healthcare [8]. However, the community element as well as collaborating across institutions/settings remain a novelty in palliative care education [9].

Although the vast majority of medical students are not exposed to palliative care clinical encounters in the community, mainly because of curriculum priorities and logistical challenges [10], studies have demonstrated the benefits of a teaching model using bus rounds in patients’ homes [11, 12].

The main purpose of this qualitative study was to capture the experiences of a select group of medical students’ following a community-based PC course.

## Methods

We conducted a qualitative study using two focus groups, with 6 and 9 participants each, at the end of the community-based PC course called “The patient at home. Insight into the reality of care”.

Focus groups are a flexible method to capture students’ opinions, perspectives and experiences using a non-directive technique that results in the controlled production of a discussion by a group of people around a topic, in this case the course. It uses the group and its interactions to help participants explore and clarify their views in ways that would be less easily accessible in one to one interviews. The strength of focus groups lies in the ‘face validity’ of the data generated.

The collective and individual responses encouraged by the focus group setting generate material that differs from other methods [13, 14]. Focus groups have been already used in education research [15–17].

A German medical faculty offered the course during the summer terms of 2018 and 2019 as an elective for palliative medicine students in the 4th to 10th semester (equivalent to 2nd to 5th year). The medical curriculum consists of 12 semesters (2 per year), which combine preclinical and clinical subjects from start to finish (‘Z-curriculum’). Interested students were selected on a ‘first come/ first served’ basis with cap at 12 participants.

### Course description

The teaching concept was based on the CBL model Patient Home Visits, in which palliative care is taught by visiting individual patients at home or inpatient hospice settings. Patients are visited by health care providers, faculty, and students [11, 18].

**Fig. 1:**
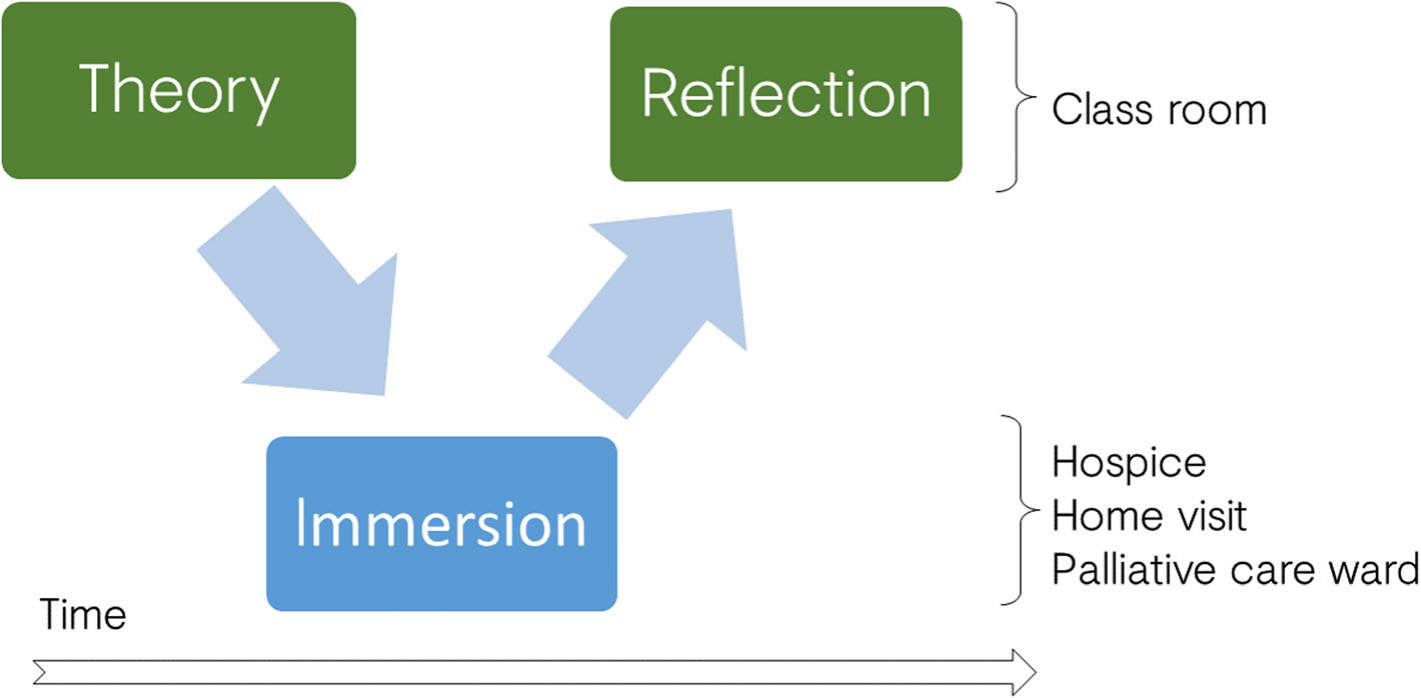
Didactic concept

We adapted the model combining visits with classroom teaching and reflection space, in order to deepen the learning experience, beyond the lived experience. The learning objectives are described in Table 1 and entail three phases (Fig. 1, Table 2) [11]:

- Phase 1 (Theory): two standard lectures: 1) Principles of PC and 2) Overview of the PC settings in Germany and challenges of care provision.
- Phase 2 (Immersion): Prior to the immersion phase, students were asked to sign a confidentiality agreement. Students were given a tour of the in-patient hospice and PC unit, where they were given the opportunity to converse with staff, family members and patients.

**Table 1:** Learning objectives

After the course the students will be able to:	
- identify the principles of palliative care in the real patient care.	
- assess advanced ill patient in their environment.	
- discuss the models of palliative care provision (home care, palliative care unit, hospice) and their relationships.	
- identify common symptoms and discuss pharmacological and non-pharmacological.

**Table 2:** Agenda for students

Session	Time	Details
**1**	2 h	Lecture: Principles of palliative medicine
	2 h	Lecture: Models of palliative care provision
**2**	2 h	Visit to inpatient hospice^a^
**3**	4 h	Home visits^b^ (subgroup) 2–3 students *
	4 h	Self-study
**4**	2 h	Unit for palliative medicine
**5**	4 h	Presentation and reflection

* It is repeated according to the number of sub groups

^a^*Inpatient hospices are independent facilities to provide holistic care for people at the last stages of an advanced disease with a life expectancy of 6 months or less* [25].

^b^*Home visits are provided by a specialized outpatient palliative care teams, which can be conducted at home, in inpatient nursing facilities and in hospices* [25].

^c^*Units for palliative medicine are a specialized part of a hospital, where critically ill patients with complex symptoms can be treated by a multi-professional team* [25].

- Since contact with seriously ill patients can generate emotional distress responses in students [19] they were closely observed throughout the course by the instructor who had been trained to respond appropriately to warning signs. Interestingly, studies have shown that patients do not experience any additional stress from the presence of students [11].
- Planning for home visits required identifying patients, contacting them in advance, and asking to participate in the study. Those who agreed had to sign a consent form allowing students visiting their homes. Six groups of three students were formed. Each subgroup of three, with the treating physician from the staff of the home-care program and the instructor, constituted a ‘car pool’.
- During the car trip to the patient’s home, students were briefed about the patient history, current treatments, and goals of the visit. Before entering the home, patients and relatives were asked to confirm their prior authorization for students to participate in the home visit.
- During the home visit, the students were asked to observe and reflect on three areas: the clinical encounter, the setting/environment, and symptom management.
- Students and faculty debriefed during the return car trip.
- Phase 3 (Period of reflection): One week after the final home visit, each student subgroup shared a summary of the home visits and presented each other one aspect of the activity, a literature review, and their own reflections. In this context, reflection is an ongoing process of evaluating, interpreting, and thinking that aims to deepen into professional, personal and emotional aspects. Additionally, the learning objectives were evaluated through direct feedback.
- Students were protected by their regular student insurance for outside activity. All participants were invited to a focus group at the end of each course. Participation in the focus groups was voluntary.

The focus groups were led by two trained researchers (TP and JW). The meetings took place in a hospital conference room, affiliated with the university. Meetings lasted approximately 90 min. Group participants were assured of confidentiality and consented to the discussion being recorded with a digital device. A semi-structured questioning covered the following topics: experience of the course, challenges, and suggestions for future courses.

Researchers followed the conversations and the issues raised, seeking clarification or details as necessary. Participants were encouraged to be honest and open, and to express their opinions about their experience, even if they were negative.

### Data analysis

Recordings were transcribed verbatim. Audio tapes were deleted after anonymized transcription in the original German, using transcriptions rules [20]. This ensures anonymity of individual participants.

A thematic analysis was conducted with the support of qualitative analysis software [21] to identify the main issues discussed by participants [22]. The transcripts were thoroughly read and encoded by the first author and each text segment related to a specific topic was assigned a code. Codes were grouped by concept groups, which were used to create categories and generate themes. The second author then scrutinized the encoded text. Any ambiguities or disagreements regarding the assignment of codes or the development of topics were resolved by consensus. Selected quotations were translated for this paper.

## Results

Fifteen students - all Caucasian German women in their 2nd to 5th year - took the course (6 in 2018 and 9 in 2019) (Table 3). All course participants joined the focus groups. The following themes emerged from the discussion:

**Table 3:** Sociodemographic and educational characteristics of participants

Characteristics	Value, n (%)a
Age (years) (mean ± SD)	23.5 (± 2.1)
Sex	
Women	15 (100%)
Point in their studies	Median 4th year (IQR = 3)
2nd year	4 (26.7%)
3rd year	2 (13.3%)
4th year	4 (26.7%)
5th year (final)	5 (33.3%)
Mandatory palliative care course	
Yes	60%
No	40%
Practical experience in palliative & hospice care	
Yes (in a palliative care unit)	2 (13.3%)

### Authenticity

The authenticity of the situations contrasted with the usual medical school experience. Although simulations with actors are a good strategy for those who have only limited opportunities to experience real end-of-life situations [23], there is no comparison with ‘real’ patient contact – especially with patients at the end of life.

A [the letter does not represent a single student]: (...) In the ‘Palli-block’ [mandatory PC course], in 7th semester [3rd year], I think you get good insights, but that is a huge difference to actually meeting a patient in the real situation. We had actor-patients in the block, but that's never as impressive as meeting a patient in the last phase of life. (5th year, 25 y.)

B: If you meet the patient in her/his own setting, you can better imagine what kind of person they are, in their own four walls. I haven't been to a hospice yet ... I found it very impressive. It was good to take this impression with me. (4th year, 23 y.)

All participants felt that the course was worthwhile both because of the knowledge gained and the personal experience.

### New knowledge and demystification

The hospice and palliative ward visits caused participants to revise previous (mis-) perceptions regarding these facilities and their functions. Although these perceptions were not explicitly recorded in the context of this evaluation, they repeatedly appear implicitly in the students’ statements and are also well-known from the literature, as well as from personal experience and individual discussions with patients and their relatives. Medical students and non-palliative health personnel consider hospices and PC units as sad, dark, and quiet places.

A: I think, it was amazingly interesting to visit the hospice... I had a completely different idea about it! It was the highlight! (5th year student, 24 y

Researcher (R): How did you imagine it?

A: I had a different picture ... darker... more hospital-like... I haven’t been in a hospice before ... I found the whole facility very impressive. (5th year, 24 y.)

B: I had also a different picture... I thought that it is run much more like a hospital... not that people have time to talk with the patient... and that they [hospices] do much more at the social level than at the medical level. (4th year, 23 y.)

This quote redirects to the next topic: the concept of PC ‘in action’.

### Palliative concept in the practice

The previous quote shows how the social dimension was recognized as a central aspect of PC in addition to – and sometimes even before – the clinical aspect, which primarily refers to treatment of physical symptoms. Additionally, the students were exposed to a world outside their daily hospital experience. They experience a patient-centered encounter, which has a ‘humanizing’ effect, and that the work is transdisciplinary. That is that we call the ‘PC concept in the practice’.

A: It was exciting for me to see what outpatient care looks like. During the Palli-block it was rather like a regular hospital… it is good to see other structures ... The Palli- network, which was presented, with so many people from different areas that work together, I found that very impressive. (5th year, 24 y.)

The participant recognized the expansion of the (practical) possibilities offered by PC. One student recalled an experience from her personal environment and how it was dealt with in her memory from a medical perspective:

A: 'Your life will end in a few weeks, we will not do anything more'... not true, there are simply so many options that you still have, communicative, but also pharmaceutical, and I like that, there is so much to learn. (5th year, 24 y.)

The range of medical procedures that can also be carried out at home became clear:

A: ... I found it very exciting how many things (procedures) are possible at home, such as the fact that palliative sedation is possible. (5th year, 30 y.)

Often, during daily hospital work, patients are reduced to cases or symptoms. Now, however, the students see the patient as a whole person with a story.

A: Also, that you can see that the patient is more than these symptoms. At home or in the hospice you can see even more of the patient, about their stories, social life... it should play a bigger role in everyday hospital work. (3rd year, 23 y.)

### Doctoring’: professional modelling

The course provides opportunities to see experienced doctors in challenging situations. How does an expert handle situation with dying patients and their families? Or young patients with fatal prognoses? Students can observe and reflect on the clinical encounter.

A: Also watching a doctor was very exciting. Palliative care doctors have a particularly kind way with patients, which is always great to witness, so I liked to learn some tricks. (5th year, 23 y.)

B: And I know from my family context, where a cancer diagnosis was just so poorly communicated, all the fears were not addressed because the doctors were unable to communicate well. I can see that there are many ways to deal with the situation and do something good for the patient. (2nd year, 22 y.)

Debriefings took place during the drive home and addressed questions that came up both before and after the home visits. These conversations facilitated transitions from theoretical knowledge to the real-life experience and the other way round (from theory into to the reality).

### Emotional experience

One of the issues the faculty considered was the emotional response that could result from face-to-face interactions with real patients.

A: For me personally I had no fears, but it was strange in part, I had to pull myself together a few times. It was very important for me to experience this situation. I can well imagine that doctors had to communicate with the patients, but they are afraid to say something wrong. (2nd year, 22 y.)

B: It was rather distressing; I needed a few days to ... I had to cope with it. But I find it ok, it would be weird, if these conversations were… as if nothing would happen. (5th year, 24 y.)

Students affect was monitored during the home visits, especially after some emotionally charged situations. The monitoring did not identify any problematic situations with the students.

Researcher: Would you have needed more support?

A: It was ok like that. We always spoke, each of us had the chance to come to you and say something. It was okay ... (3rd year, 22 y.)

B: Well, we also reflected directly in the car. That was helpful! (4th year, 23 y.)

Apparently, PC is unpopular among medical students. Participants almost needed to justify themselves to their fellow students.

A: They [my fellows] said, how could you have looked at that! (…)... many could not understand that it is another level, that the focus lies on doing the best possible until the end... as stupid as it sounds. (2nd year, 21 y.)

### Structure of the course

Students gave the course structure positive evaluations. The interactive lectures were also given high marks.

Preparatory support for reflection (medical encounter, setting and symptoms) during the home visit was perceived as helpful:

It was quite good again to have prepared, to learn what to look for, because so much was happening above and beyond the conversations and there were simply many new impressions... And then… to identify a topic that you find especially interesting in the whole scenario. (4th year, 23 y.)

The reflection at the end finally closed the circle:

It was great to have time to reflect about the patient. What we have done today [reflection], I found it really good, because you can process things a bit and you can sort it and put it in order again. (5th year, 24 y.)

### Curriculum requirements

The participants recognized the need for this course and identified the deficit in the curriculum, which does not include contact with actual PC patients. As future physicians, the students considered it important to deal with the last days of life. The course offers this opportunity in a safe and protected environment, with room for reflection.

## Discussion

The course both provided students with knowledge and gave them an opportunity to experience and reflect on how they handled challenging situations. Course participants identified four main areas of relevance: authenticity, demystification of the concepts of hospice and PC through practical application of theoretical knowledge, and watching a role model interact with seriously ill patients and conduct difficult conversations.

The students identified PC principles as holistic, patient-centered, multidisciplinary and aimed at improving the quality of life for people in a difficult situation. They also learned about communication skills and pharmacological based strategies to achieve this goal.

Participants saw the lecturers as role models in patient care, in family and patient communications, and in symptom assessment and treatment.

PC’s humanistic focus on the patient as an individual, rather than as a representative of their illness, became clear during the course. Students learned to focus on how to involve patients in goals of care, respect their perspectives and differences, and educate them about the disease trajectory and treatment [24].

Participants’ sense that questions were welcome resulted in interactions that developed both in class and during patient visits. In particular situations, some students felt embarrassed or powerless due to changes in the relationship – the patient rather than the clinician was in control and had the requisite expertise regarding his or her condition.

Even distressing experiences were perceived as part of the learning process rather than negatively, which is why the lecturers’ openness to discuss and debrief in the car was important. Students were confronted with emotionally ‘charged’ situations that could have been distressing. Strategies such as debriefing immediately after the situation, reflecting on what had occurred and offering opportunities to discuss with the lecturers, kept this burden manageable.

The course’s usefulness was independent of the participant’s previous experience. Ultimately each participant was able to learn/experience something, regardless of the course/ semester they were enrolled in. Unfounded prejudices against hospice or PC were corrected and possibilities of caring in difficult situations were re-evaluated.

Our course ‘The patient at home. Insight into the reality of care’ was a successful adaptation of the original model. It did demonstrate validity and appropriateness to be used and looked at elsewhere in the curriculum and have an evidence basis to be used in the community palliative care teaching.

## Limitations

Participants were highly motivated, which could have biased the positive evaluation. The fact that the lecturers moderated the focus group could also have influenced the free reviews of the participants. Students and patients shared the same ethnic and cultural background, therefore we were unable to assess the effect of these factors.

Project implementation required logistical planning for participating students and adaptation. In the original concept, participants rode on a bus, which was not possible in our setting. We replace it with a ‘car pool’.

The experience was remarkable for the students, which can possibly result in a very positive rating of the course. It would be interesting to know if the learning is sustainable over time and if it impacted the patient care of these future physicians.

## Conclusions

Community-based learning in outpatient settings and classroom teaching are very different approaches to learning and their combination enriches the learning process from theory to practice and from everyday practice to teaching. A key pedagogical element is to enable students to apply what they have learned in the classroom and to reflect on their experience. The risk is that, if lectures are not followed up with experience and reflection, students miss the human aspects of the experience. It is necessary to integrate this kind of teaching in educational PC programs.

Students’ apprehension of the patient and their family beyond the medical relationship allowed them to better appreciate the holistic claim of PC. Bringing students directly from the hospital to the patients in their homes shows the benefits of an integrated healthcare system. Students realize the possibilities and limits of the different PC models of service provision.

Health care educators should consider evaluating and changing their clinical teaching environment, and exploring community-based learning as the ‘Patient Home Visits’ model, adapting it to the own needs. This concept could, conceivably, be transferred to other medical faculties and subjects. Further analysis of the topics and additional recording of patient and faculty would be interesting for future projects.

## Data Availability

The datasets analysed during the current study available from the corresponding author on reasonable request.
